# General Type-2 Fuzzy Sugeno Integral for Edge Detection

**DOI:** 10.3390/jimaging5080071

**Published:** 2019-08-16

**Authors:** Gabriela E. Martínez, Claudia I. Gonzalez, Olivia Mendoza, Patricia Melin

**Affiliations:** 1 Division of Graduate Studies and Research, Tijuana Institute of Technology, Tijuana 22414, Mexico; 2 School of Engineering, Autonomous University of Baja California, Tijuana 22390, Mexico

**Keywords:** Sugeno integral, fuzzy edge detection, general type-2 fuzzy sets

## Abstract

A type-2 fuzzy edge detection method is presented in this paper. The general process consists of first obtaining the image gradients in the four directions—horizontal, vertical, and the two diagonals—and this technique is known as the morphological gradient. After that, the general type-2 fuzzy Sugeno integral (GT2 FSI) is used to integrate the four image gradients. In this second step, the GT2 FSI establishes criteria to determine at which level the obtained image gradient belongs to an edge during the process; this is calculated assigning different general type-2 fuzzy densities, and these fuzzy gradients are aggregated using the meet and join operators. The gradient integration using the GT2 FSI provides a methodology for achieving more robust edge detection, even more if we are working with blurry images. The experimental evaluations are performed on synthetic and real images, and the accuracy is quantified using Pratt’s Figure of Merit. The results values demonstrate that the proposed edge detection method outperforms other existing algorithms.

## 1. Introduction

The edge detection process is widely used in pattern recognition and computer vision systems because it is helpful in obtaining satisfactory results when applied in the preprocessing phase. Nevertheless, the process of finding the edges is not easy, especially if the image is blurry or distorted by noise; these phenomena frequently occurs when the image is captured by any acquisition hardware, factors like the distance, quality, and resolution of cameras, environment, and illumination variation tend to produce images with ambiguous or incomplete data. The issue of determining what is an image edge and what is not becomes more critical by the fact that edges are partially distorted or hidden.

In order to solve this issue for image edge detection, in recent years, various approaches that involve soft computing methods have been put forward, including the principles of fuzzy set theory, which is one of the main topics in this work. The aim of implementing the fuzzy reasoning proposal by Zadeh [1] for these kinds of tasks is because this is useful in modeling and approximating ambiguous or vague information, which is found in these problems.

Fuzzy techniques for edge detection have gained importance because they offer a good alternative to enhance the accuracy in the edge detection process. We can mention some important contributions for edge detection using type-1 (T1), interval type-2 (IT2), interval-valued and general type-2 (GT2) fuzzy sets (FS). Law et al. [2] and Russo [3,4] introduced an approach to edge detection applying fuzzy reasoning; in [3] fuzzy edge detection was applied to noisy images obtaining satisfactory results. Liang et al. [5] presented a competitive fuzzy edge detection method. Wu et al. [6] presented an edge detection algorithm with a multilevel fuzzy approach for blurry images. Bustince et al. [7] and Barrenechea et al. [8] introduced a new method for edge detection using interval-valued fuzzy theory. Mendoza et al. [9] and Melin et al. [10] proposed some image edge detectors methodologies implementing IT2 FS, and these achieved better results than the T1 fuzzy edge detectors. Molina et al. [11] presented a method to generate fuzzy image edge detection where the image gradients are transformed into membership degrees implementing parametric membership functions. Melin et al. [12] and Gonzalez et al. [13] presented interesting fuzzy edge detection approaches using GT2 FS theory, and these improved the edges compared to existing T1 and IT2 fuzzy edge detection methods.

As was mentioned previously, this paper aims at applying the general type-2 fuzzy Sugeno integral (GT2 FSI) [14] in aggregating the image gradients and to produce more robust edge detection. In the methodology, the image gradients and fuzzy densities are fuzzified using general type-2 membership functions including other operators, such as the meet and join which are applied in aggregating the fuzzy information sources. This procedure has the advantage of handling higher levels of vagueness or uncertainty that could be found in the images. 

The GT2 FSI is an extension of the interval type-2 fuzzy Sugeno Integral (IT2 SI) [14] and the conventional Sugeno Integral [15]. Melin et al. [16] proposed a method to integrate modular neural networks using the Sugeno Integral; this was applied for time series forecasting. In addition, Melin et al. [17] also introduced an approach using the IT2 SI to aggregate the outputs of a face recognition system based on modular neural networks. In both proposals, the authors present satisfactory results when compared to other aggregation methods. In general, information aggregation is an important and necessary process to perform any decision-making task; nevertheless, this can be applied to any problem when we need to aggregate different information sources or different criteria. Implementing the GT2 FS theory [18,19,20] is possible to generate powerful aggregation techniques with the capability of handling the uncertainty information present in any image processing system [21,22,23]; being this one of the main motivations in developing this proposal.

The remainder of this paper is structured as follows. The methodology to obtain the image gradients is explained in Section 2. Section 3 presents some basic definitions about the fuzzy measures and the Sugeno integral, which are essential concepts for this work. Section 4 illustrates in detail the General type-2 fuzzy Sugeno integral approach. Section 5 presents the methodology to generate the fuzzy edge detection method and technically describes how the image gradients are aggregated using the GT2 FSI. Section 6 gives the experimental simulations when the proposed method is applied to synthetic and benchmark image databases and presents the comparison of results against other approaches. Finally, Section 7 offers some conclusions of this work, significant remarks, and future works.

## 2. Morphological Gradient Approach for Edge Detection

Edge detection is an essential phase in image processing and computer vision systems; especially, in applications for feature extraction or detection of significant elements found in the image. This process may simplify and reduce the amount of data to be processed; it can be applied as a filter to discard the irrelevant characteristics and to preserve the essential properties of the image.

For humans, it is easy to recognize an object with little information about it or only its edges, it is expected that for image recognition systems will be easier to learn with fewer image features and achieve better recognition results.

In the literature, we can find various ways to perform the process of edge detection, where the most used techniques are focused on obtaining the gradient by calculating the first derivative of the image. There are several methods based on this approach; the best known are the Roberts, Sobel, and Prewitt operators [24,25], these apply a convolution process that uses a kernel to approximate the image gradient and returns the values of the first derivative only for the horizontal and vertical direction. 

**Figure 1:**
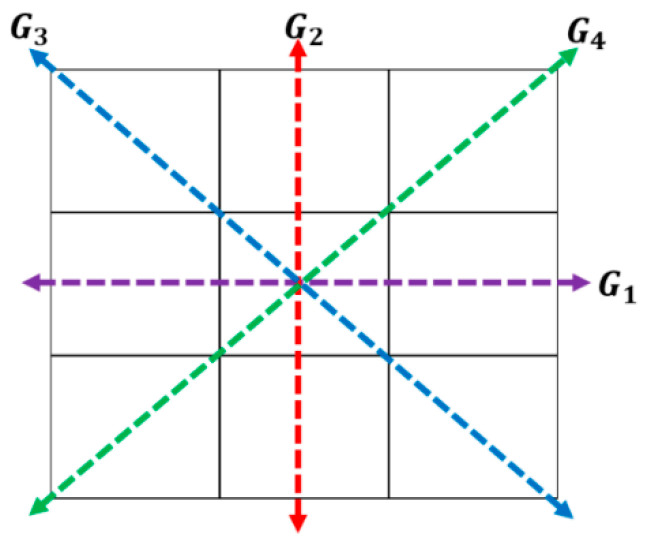
Gradients direction $G_{i}$.

In this paper, we are proposing to use the morphological gradient (MG) approach, which consists of calculating the first derivative, but for the four angles or directions of the image; i.e., vertical, horizontal and the two diagonals (0, 45, 90 and 135 degrees), as is shown in Figure 1, and indicated by the variables G_1_, G_2_, G_3_, and G_4_ respectively. These image gradients are calculated as follows.

**Figure 2:**
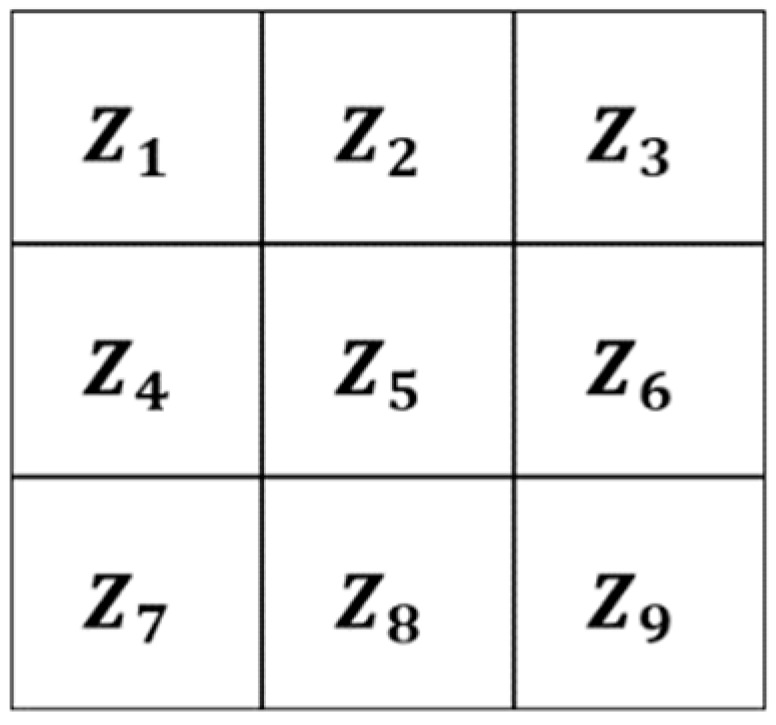
Coefficients $Z_{i}$.

The variable G_i_ (for i = 1…4) is used to represent the possible directions of the gradients (edges), and these are calculated with Equation (2) by applying a 3 × 3 matrix. In Equation (2), z_i_ represents the coefficients of matrix positions (Figure 2) and, these are obtained with Equation (1); where *f* represents the image, x-axis the columns and y-axis the rows. Finally, the possible edge value E is integrated by using Equation (3) [10], [26,27].

(1) $$\begin{array}{l} z_{1}=f\left(x-1,y-1\right)\;\;z_{2\;}=f\left(x,y-1\right)\\ z_{3}=f\left(x+1,\;y-1\right)\;\;z_{4}\;=f\left(x-1,y\right)\\ z_{5\;}=f\left(x,y\right)\;\;\;\;\;\;\;\;\;\;\;\;\;\;\;\;\;z_{6}\;=f\left(x+1,\;y\right)\\ z_{7}=f\left(x-1,y+1\right)\;\;z_{8\;}=f\left(x,y+1\right)\\ z_{9}=f\left(x+1,\;y+1\right) \end{array}$$

(2) $$G_{1}=\;\sqrt{{\left(z_{5\;}-\;z_{2}\right)}^{2\;}+\;{\left(z_{5\;}-\;z_{8}\right)}^{2\;}}\;G_{2}=\;\sqrt{{\left(z_{5\;}-\;z_{4}\right)}^{2\;}+\;{\left(z_{5\;}-\;z_{6}\right)}^{2\;}}\;G_{3}=\;\sqrt{{\left(z_{5\;}-\;z_{1}\right)}^{2\;}+\;{\left(z_{5\;}-\;z_{9}\right)}^{2\;}}\;G_{4}=\;\sqrt{{\left(z_{5\;}-\;z_{3}\right)}^{2\;}+\;{\left(z_{5\;}-\;z_{7}\right)}^{2\;}}$$

(3) $$E=G_{1}+G_{2}+G_{3}+G_{4}$$

## 3. The Sugeno Integral and Fuzzy Measures

There exist operators that allow us to perform mathematical operations on elements that form a collection, and usually, the overall result is a numerical value. 

In recent years, fuzzy integrals and fuzzy measures have become popular in various areas of research on aggregation operators, so it is interesting to apply these operators in different study cases to analyze their behavior. Usually when an aggregation process is used, the computer systems that need to combine or fuse information require preliminary processing. In particular, for the case of the morphological gradient edge detector, the information sources are the gradients calculated in the four directions. In general, it is possible to distinguish among the process of aggregation, information integration, and information fusion.

Fusion consists of combining information, for example, combining degrees of satisfaction of a user. The integration uses information from various sources for a particular purpose, for example, taking the online information base to decide the best destination for a trip. Aggregation operators combine information when mathematically formalized, for example, the arithmetic mean. For a better understanding, this Section defines the Sugeno measures and Sugeno Integral.

### 3.1. Sugeno Measures

In the literature, we can find the term λ-measures, defined as a particular type of monotonic measures [28]. If we have a universe of discourse X and a nonempty family D of subsets of X, a monotone measure μ on 〈X, D〉 is a function of the form μ: D → [0,∞]. It is assumed that the universal set X is finite and that D = P(X). That is, the monotonic measures of concern are sets of functions μ: P(X) → [0, 1].

**Definition** **1.**  *A monotonic set measure μ on space X is a mapping μ: P(X) → [0, 1] such that the following properties hold [29,30]:*

*1)*  *μ(∅)= 0*
*2)*  *μ(X) = 1*
*3)*  *For all B, C ∈ P(X), if B ⊆ C, then μ(B) ≤ μ(C)*

A monotonic measure is established as follows: Also called fuzzy measure (FM) and denoted by μ, is represented by μ: 2^x^ in the interval [0,1] for a finite dataset X = {x_1_, x_2_, …, x_n_}, and that must comply with the conditions shown below:

1) μ(∅) = 0 and μ(X) = 1.
2) Given B, C ∈ 2^x^, if B ⊂ C then μ(B) ≤ μ(C) (monotonicity property). In this case, B and C represent subsets of the set X.

If μ(B) and μ(C) are two disjoint sets, we can still reconstruct the degree μ(B ∪ C). The reconstructed value is no longer the sum μ(B) + μ(C), but a slightly more complex expression. A Sugeno measure is a λ-fuzzy or also called fuzzy measure if it complies with the option 1 of addition for some −1 < λ.

μ(B ∪ C) = μ(B) + μ(C) + λ μ(B)μ(C)(4)

Equation (4) is commonly known as the λ-rule. When X represents a finite set and each element is assigned a fuzzy density (FD) μ(x) for all x of the set X, these represent the relevance of each one of the data sources. It should be noted that the importance of a subset of data from a set B used to give a solution to a problem is determined by the fuzzy measure [31]. The value of μ(B) for each of the B ⊂ *X* is calculated by the recurrent application of the λ-rule, and this parameter can be expressed by using Equation (5).

(5) $$\mu \left(B\right)=\;\left[\frac{\prod_{x\in B}\left(1+\lambda \mu \left(\left\{x\right\}\right)\right)}{\lambda }\right]$$

Once the FD μ({x})  is defined, using the constraint μ(x) = 1, we can calculate the λ value so that when applying Equation (5) we obtain Equation (6).

(6) $$\lambda \;+\;1\;=\prod_{i=1}^{n}\left(1\;+\;\lambda \mu \left(\left\{x_{i}\right\}\right)\right)$$

An element that characterizes this type of measure is the lambda parameter (λ), and this can be calculated with Equation (6) when the FD is assigned.

One of the important works of Sugeno was to demonstrate that this polynomial function is numerically easy to solve and that its root is greater than −1. The property of Equation (6) allows decreasing the free parameters numbers from 2^n^ − 2 to *n* since it stipulates the *n* different densities [32]; the lambda parameter (λ) is calculated based on the Theorem 1 [33]:

**Theorem** **1.**  *Let μ({x}) > 0 in at least two elements of X, and 1 > μ({x}) for all x that belong to the set X. Then, the unique parameter λ is calculated with Equation (6) as follows:*

- *If $\underset{x\in X}{\sum }\mu \left(\left\{x\right\}\right)<1$, then λ is a value in (0,∞).*
- *If $\underset{x\in X}{\sum }\mu \left(\left\{x\right\}\right)=1$, then λ = 0;*
- *If $\underset{x\in X}{\sum }\mu \left(\left\{x\right\}\right)>1$, then λ is a value in (−1, 0).*

If μ is a λ-FM, using Equation (5) μ(B_i_) is calculated recursively rearranging μ({x}) and the sets X in descending order taking as reference the values of the set X [34,35].

### 3.2. Sugeno Integral

This integral can be interpreted from two points of view: decision making under uncertainty and the decision making of multiple criteria. Sugeno defined the fuzzy integral term with respect to fuzzy measurements (FM or λ-FM) as non-linear functions [15]. The "*maxmin*" operators are generalized by Equation (7); this can be understood as finding the highest degree of likeness with the expected value and the target.

(7) $$Sugeno_{\mu }\left(x_{1},x_{2},\ldots ,x_{n}\right)=\underset{i=1..n}{\max}\left(\min\left(D\left(x_{\sigma \left(i\right)}\right),\;\mu \left(A_{\sigma \left(i\right)}\right)\right)\right)$$

where $\;x_{\sigma \left(i\right)}$ determine that the data $D(x_{\sigma \left(i\right)})$ need to be permuted as $0\leq D\left(x_{\sigma \left(1\right)}\right)\leq D\left(x_{\sigma \left(2\right)}\right)\leq \ldots \leq D\left(x_{\sigma \left(n\right)}\right)\leq 1$, and $A_{\sigma \left(i\right)}=\left\{\sigma \left(i\right),\ldots ,\sigma \left(n\right)\right\}$.

Any problem in which X = {x_1_, ..., x_n_} is considered as a finite set, can be solved using the Sugeno integral. In Algorithm 1, the steps to evaluate the Sugeno integral (SI) are presented.

**Algorithm 1:** Sugeno integral algorithm.**Input:***n* represents the sources number; {x_1_, x_2_, …, x_n_} denotes the information and the fuzzy densities of the information sources are M_1_, M_2_, …, M_n_ ∈ (0,1).**Output**: Sugeno integral h(σ(*x*_1_), σ(*x*_2_), …, σ(*x_n_*)).**1:** Estimate λ using the equation $f\left(\lambda \right)=\left\{\overset{n}{\underset{i=1}{\prod }}\left(1+M_{i}\left(x_{i}\right)\lambda \right)\right\}-\left(1+\lambda \right)$
**2:** The variable x_i_ must be fuzzify using.$D_{i}=\left\{\left(x_{,}\mu_{Di}\left(x\right)\right)|x\;\epsilon \;X\right\},\;\mu \{D_{i}\}\left(x\right)\in [0,1]$**3:** Reordered with base on D(x_i_) all the M_i_ in descending order.**4:** Determine the FM for all *D_i_* using Equation (4).**5:** Calculate Sugeno integral with Equation (7).**6:** OUTPUT Sugeno integral.

## 4. General Type-2 Fuzzy Sugeno Integral

In recent years, there has been a significant increase in the fuzzy research area, especially in interval type-2 fuzzy logic and more recently in general type-2 fuzzy logic. The main idea of working with higher orders or types of fuzzy logic is to build models of uncertainty more robust, so it is expected that better results will be obtained when making use of the general type-2 fuzzy logic. Based on the above, it is of great interest to combine the fuzzy Sugeno integral and general type-2 fuzzy systems to achieve better handling of uncertainty in the process of aggregation of various information sources.

When the Sugeno integral is used as an operator to make decisions, the number of information sources *n* and the fuzzy densities M(x_i_) ∈ (0,1) must be defined for each information source D(x_i_). The series of steps used to extend the Sugeno integral applying the GT2 FS are described below [14].

**Figure 3:**
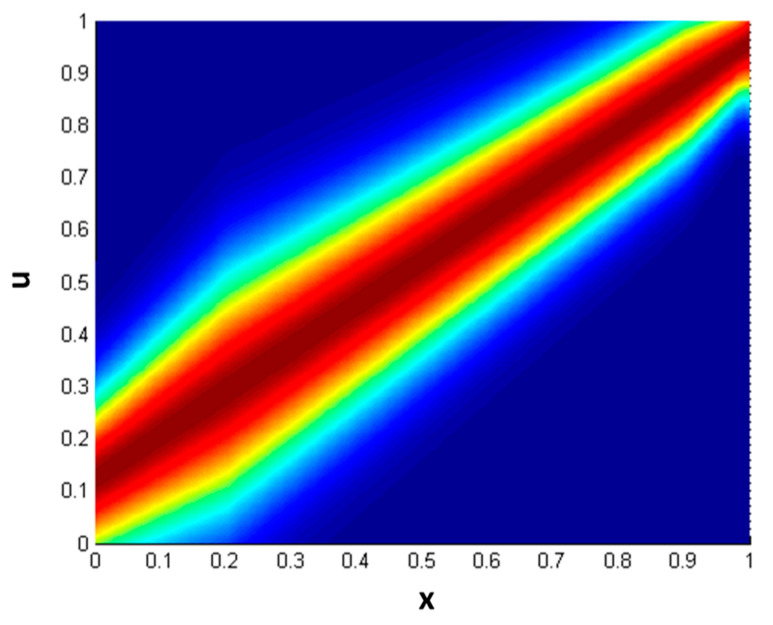
Generalized membership function trigausstype-2.

1) The first step is to evaluate each D(x_i_) and M(x_i_) using a GT2 membership function (MF) that is expressed by Equation (8), and calculated using Equation (9). In Equation (8), “*trigausstype2*” represents a Gaussian GT2 MF with uncertainty in the mean, where *x* denotes the domain of the primary MF and *u* is the partition for the secondary MF. Figure 3 illustrates a representation of this GT2 MF.
  (8) $$\tilde{\mu }\left(x,u\right)=trigausstype2\left(x,u,\left[a_{1},b_{1},c_{1},a_{2},b_{2},c_{2},\rho \right]\right)$$
  (9) $$\tilde{\mu }\left(x,u\right)=exp\left[-\frac{1}{2}{\left(\frac{u-px}{\sigma_{u}}\right)}^{2}\right]$$
  In Equation (9), the parameter *σ_u_* is calculated with Equation (10). Where *ρ*, is an uncertainty fraction that is added in the support of the secondary MF, *δ* is obtained by Equation (11), *p_x_* with Equation (12)*,* the variables *a*, *b* and *c* represent the parameterization of the MF and are determined using Equation (13).
  (10) $$\sigma_{u}=\frac{1+\rho }{2\sqrt{3}}\delta +\varepsilon$$
  (11) $$\delta =\;\bar{\mu }\left(x\right)-\underline{\mu }\left(x\right)$$
  (12) $$px\;=max\left(min\left(\frac{x-a}{b-a}\;,\frac{c-x}{c-b}\right),0\right)$$
  (13) $$a=\;\frac{a_{1}+a_{2}}{2},\;\;\;\;\;b=\;\frac{b_{1}+b_{2}}{2},\;\;\;\;\;\;c=\;\frac{c_{1}+c_{2}}{2}$$
2) The next step is obtain the alpha cuts (α_i_) for all the D(x_i_) and M(x_i_) to calculate $\mu (M_{iL\alpha_{i}}\left(x_{i}\right)$), $\mu (M_{iR\alpha_{i}}\left(x_{i}\right)$) and $\mu (D_{L\alpha_{i}}\left(x_{i}\right)$), $\mu (D_{R\alpha_{i}}\left(x_{i}\right)$).
3) After calculating the α_i_ cuts, based on Equation (4) the next thing is estimate lambda (λ) and the alpha cuts (α_i_) for the lambda left ($\lambda_{L}$) and lambda right ($\lambda_{R}$) applying Equations (14) and (15).
  (14) $$f\left(\lambda_{L\alpha_{i}}\right)=\left\{\overset{n}{\underset{i=1}{\prod }}\left(1+M_{iL\alpha_{i}}\left(x_{i}\right)\lambda_{L\alpha_{i}\;\;}\right)\right\}-\left(1+\lambda_{L\alpha_{i}\;\;}\right)$$
  (15) $$f\left(\lambda_{R\alpha_{i}}\right)=\left\{\overset{n}{\underset{i=1}{\prod }}\left(1+M_{iR\alpha_{i}}\left(x_{i}\right)\lambda_{R\alpha_{i}\;\;}\right)\right\}-\left(1+\lambda_{R\alpha_{i}\;\;}\right)$$
  Once $\lambda_{L\alpha_{i}}$ and $\lambda_{R\alpha_{i}}$ values are obtained, the next step is determine $\mu_{L\alpha_{i}}\left(A_{i}\right)\;$ and $\mu_{R\alpha_{i}}\left(A_{i}\right)$ by using Equations (16)–(19).
  (16) $$\mu_{L\alpha_{i}}\left(A_{1}\right)=\mu_{L\alpha_{i}}\left(x_{1}\right)$$
  (17) $$\mu_{L\alpha_{i}}\left(A_{i}\right)=\mu_{L\alpha_{i}}\left(x_{i}\right)+\;\mu_{L\alpha_{i}}\left(A_{i-1}\right)+\lambda_{L\alpha_{i}}\mu_{L\alpha_{i}}\left(x_{i}\right)\mu_{L\alpha_{i}}\left(A_{i-1}\right)$$
  (18) $$\mu_{R\alpha_{i}}\left(A_{1}\right)=\mu_{R\alpha_{i}}\left(x_{1}\right)$$
  (19) $$\mu_{R\alpha_{i}}\left(A_{i}\right)=\mu_{R\alpha_{i}}\left(x_{i}\right)+\;\mu_{R\alpha_{i}}\left(A_{i-1}\right)+\lambda_{R\alpha_{i}}\mu_{R\alpha_{i}}\left(x_{i}\right)\mu_{R\alpha_{i}}\left(A_{i-1}\right)$$
4) Taking as a reference the Equation (7), is necessary to calculate the Sugeno integral for $\alpha_{i}$ using Equation (20).
  (20) $$h({\tilde{\sigma }}_{\alpha_{1}},\;{\tilde{\sigma }}_{\alpha_{2}},\;\ldots ,\;{\tilde{\sigma }}_{\alpha_{n}})={⊔}_{I=1}^{n}\left(⨅\left[h_{L\alpha_{1}},h_{R\alpha_{1}}\right],⨅\left[h_{L\alpha_{2}},h_{R\alpha_{2}}\right],\ldots ,⨅\left[h_{L\alpha_{n}},h_{R\alpha_{n}}\right]\right)$$
  In Equation (20), each ${\tilde{\sigma }}_{\alpha_{i}}$ represents an interval of the form Equation (21).
  (21) $${\tilde{\sigma }}_{\alpha_{i}}={⊔}_{i=1}^{n}\left(⊓\left(\left[\mu (D_{L\alpha_{i}}\left(x_{i}\right)),\mu_{L\alpha_{i}}\left(A_{i}\right)\right],\left[\mu (D_{R\alpha_{i}}\left(x_{i}\right)),\mu_{R\alpha_{i}}\left(A_{i}\right)\right]\right)\right)$$
  Equation (21) defines the composition; the symbol ⊓ represents the meet operator of the GT2 FS and ⊔ is used to represent the join.
5) To obtain the GT2 FSI, finally, we calculate the supreme of the ${\tilde{\sigma }}_{\alpha_{i}}$ using Equation (22).
  (22) $$h=\begin{array}{c} sup\\ i \end{array}\left(h\left({\tilde{\sigma }}_{\alpha_{i}}\right)\right)$$
  The general type-2 fuzzy Sugeno integral is summarized in Algorithm 2.

**Algorithm 2:** General type-2 fuzzy Sugeno integral algorithm**Input:***n* represents the sources number, D(x_i_) denotes the information and M(x_i_) the FD in the domain (0,1).**Output:** General type-2 Sugeno integral *h*.**1:** Estimate the M(x_i_) and D(x_i_) using the function $\tilde{\mu }\left(x,u\right)=trigausstype2\left(x,u,\left[a_{1},b_{1},c_{1},a_{2},b_{2},c_{2},\rho \right]\right)$
**2**: Determine the alpha cuts (α_i_) for D(x_i_) and M(x_i_)$$\mu (M_{iL\alpha_{i}}\left(x_{i}\right)),\mu (M_{iR\alpha_{i}}\left(x_{i}\right))$$$$\mu (D_{L\alpha_{i}}\left(x_{i}\right)),\mu (D_{R\alpha_{i}}\left(x_{i}\right))$$**3**: Calculate λ and the *α_i_* cuts for $\lambda_{L}$ and $\lambda_{R}$ using Equations (14) and (15).**4**: Determine the FM $\mu_{L\alpha_{i}}\left(B_{i}\right)\;$ and $\mu_{R\alpha_{i}}\left(B_{i}\right)$ applying Equations (16)–(19).**5**: Calculate the Sugeno integral $h\left({\tilde{\sigma }}_{\alpha_{i}}\right)$, for all the α*_i_* with Equation (20).**6**: Finally, calculate the GT2SI 

The proposed general type-2 fuzzy Sugeno integral can be applied in any study case where information is combined, that can be formalized mathematically.

## 5. Edge Detection Using General Type-2 Fuzzy Sugeno Integral

**Figure 4:**
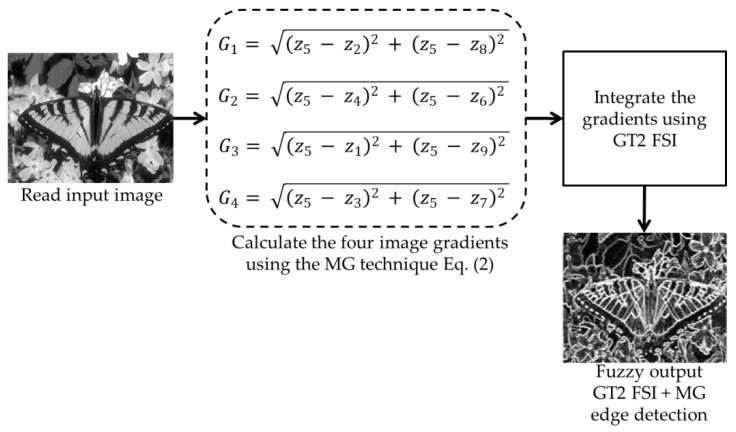
General diagram for general type-2 fuzzy Sugeno integral (GT2 FSI) + morphological gradient (MG) edge detection.

The edge detection process is widely used in pattern recognition and computer vision, nevertheless, the process to determine the edges is not easy, the issue of determining what is and what is not an image edge is made more critical by the fact that edges in many cases are partially distorted or hidden. The aim of this work is performing the aggregation process to combine the image gradients obtained by the MG edge detector, which was explained in Section 2 with FSI. For the fuzzy aggregation, the Sugeno integral combined with the general fuzzy sets operators GT2 FSI is used (detailed in Section 4). The fuzzy densities of the Sugeno integral associated with each of the gradients, and the general type-2 fuzzy systems allow the handling of uncertainty associated with the information sources to be carried out, so it is expected that by applying the proposed method, the edge detection should be more robust than applying any of the traditional methods. A general diagram about this methodology is in Figure 4. 

**Figure 5:**
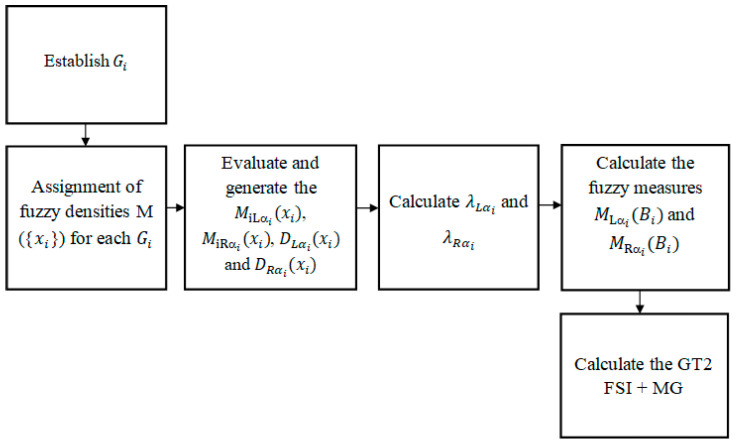
Integration of the image gradients by using GT2 FSI + MG.

**Figure 6:**
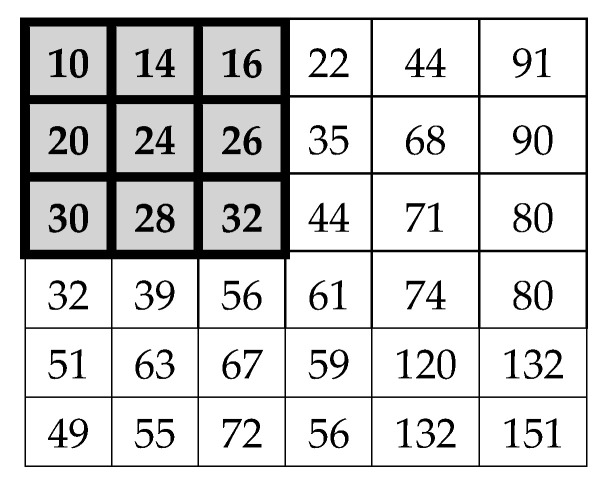
Numerical representation of an image.

The steps for integrating the gradients based on GT FSI are in Figure 5 and explained below:

1. Establish G_i_: If we have the numerical representation of an image like the one in Figure 6, and we select coefficients of the matrix positions 3 × 3 (as in Figure 1) to calculate the morphological gradient method by applying Equations (1)–(3) we obtain:
  $$G_{1}=\;\sqrt{{\left(24-\;14\right)}^{2\;}+\;{\left(24-\;28\right)}^{2\;}}\;=\;10.77$$
  $$G_{2}=\;\sqrt{{\left(24-\;20\right)}^{2\;}+\;{\left(24-\;26\right)}^{2\;}}\;=\;4.47$$
  $$G_{3}=\;\sqrt{{\left(24_{\;}-\;10\right)}^{2\;}+\;{\left(24-\;32\right)}^{2\;}}\;=\;16.12$$
  $$G_{4}=\;\sqrt{{\left(24-\;16\right)}^{2\;}+\;{\left(24-\;30\right)}^{2\;}}\;=\;10$$
  E= 10.77 + 4.47 + 16.12 + 10 = 41.36 ≈ 41
  So, the central pixel of the selected sub-matrix is replaced by the obtained value 41. However, if the proposed method is used, the G_i_ gradients are first calculated, which represent the sources of information; in this case, the normalized values are G = [0.1077, 0. 447, 0. 1612, 0.10]. For this test, five alpha cuts in the points α = [0.2, 0.4, 0.6, 0.8, 0.99] were defined for the general type-2 fuzzy system. Once obtaining the G_i_ gradients, the next step is to assign the fuzzy densities and continue with the following steps as explained below.
2. Assignment of fuzzy densities M({x_i_}) for each G_i_*:* The relevance or importance of a particular source is determined by *M({x_i_})*, thus, the information sources must be associated with one; in this case, the fuzzy densities were arbitrarily assigned. In this case, a fuzzy density M = [0.2, 0.2, 0.2, 0.2] was assigned to each of the information sources.
3. Evaluate and generate the $M_{i{L\alpha }_{i}}\left(x_{i}\right)$, $M_{i{R\alpha }_{i}}\left(x_{i}\right)$, $D_{L\alpha_{i}}\left(x_{i}\right)$ and $D_{R\alpha_{i}}\left(x_{i}\right)$: The next step is to evaluate each gradient (information sources) and fuzzy density with a general membership function using Equation (9) and generate a left and right interval for each alpha cut (see Table 1 and Table 2).
4. Calculate $\lambda_{L\alpha_{i}}$ and $\lambda_{R\alpha_{i}}$: The next thing is to determine the left and right lambda values for each alpha cut using the bisection method applying Equations (14) and (15), the results can be seen in Table 3.
5. Calculate the fuzzy measures $M_{{L\alpha }_{i}}\left(B_{i}\right)$ and $\;M_{{R\alpha }_{i}}\left(B_{i}\right)$: After that, the fuzzy measures associated with each information source are calculated (see Table 4) to perform the aggregation process using Equations (16)–(19).
6. Calculate the GT2 FSI + MG: Using the meet and join operators of the general fuzzy sets, the gradients are integrated applying the Sugeno integral with Equation (20) and (22); in this case, for each alpha cuts we obtain a solution interval. When applying the Sugeno integral, the minimum of $D_{L\alpha_{i}}\left(x_{i}\right)$, $\mu_{L\alpha_{i}}$ and $D_{R\alpha_{i}}\left(x_{i}\right)$, $\mu_{R\alpha_{i}}$ is calculated, getting the results of Table 5.

Finally, the minimum is applied to each of the left and right information sources with respect to each alpha cut, obtaining an interval for each of the alpha cuts, which can be observed in Table 6.

In the case of the proposed model, the max of each interval is calculated, obtaining as a final result [0.1447, 0.3839]; then the supreme is calculated at the interval using Equation (22), and a result of the GT2 FSI + MG is obtained by 0.3839 ≈ 0.38. Using the proposed method, the central pixel of the selected sub-matrix (see Figure 1) is replaced by the obtained value 0.38. We can notice that the final value obtained by the GT2 FSI + MG was 0.3839, this differs from the value calculated by the traditional MG that was of 41.36 (see step 1); therefore, the proposed method could be useful to determine in a more accurate way if the gradient represents an edge of the image. The latter is due to the property that the GT2 FSI + MG method has in the handling of uncertainty.

One of the main drawbacks that we have when obtaining an interval, is to determine which of the values represents the best solution. For this work, one of the approximations that we use is to calculate the average of each one of the intervals and, in the end, to select the supreme of all of them to represent the solution set expressed in Equation (22). For this particular edge detection application, the main goal was to determine whether the pixel corresponds to an edge.

## 6. Simulation Results

The results of the proposed fuzzy edge detection method are presented below, the implementation of which consists in the integration of the image gradients using the integral Sugeno combined with the GT2 FS operators.

In this section, two tests are presented; in the first test, a database of synthetic images was used, which can be seen in Table 7. In the second test, the proposed edge detector was implemented in a benchmark database of image segmentation and boundary detection (BSDS) [36] that we can found in Table 8. In both tables, we can observe the original images used to test the proposed edge detector, together with their reference images (ground true), these reference images are necessary to measure the edge detection accuracy. Additionally, to test the effectiveness of the GT2 FSI + MG method, several edge detectors were analyzed as the Morphological gradient method, the MG using the Sugeno integral (SI + MG) and MG combined with Sugeno integral and the operators of type-2 fuzzy systems (IT2 SI + MG).

For the first test, the traditional morphological gradient edge detection method was applied in both image databases (Table 7 and Table 8). The visual results of the edge detection for the synthetic images database are shown in Table 9; where the last column represents the value of the fuzzy density associated with each gradient. For the first two images, an FD = 0.6 was assigned for each gradient, while for the last three images an FD = 0.2 was used.

To evaluate the efficiency of the proposed edge detection method, the metric based on the “Figure of merit” of Pratt’s (FOM) [37,38,39] was used. FOM values are between 0 and 1 (If the result of the FOM is 1 or very close to 1, this means that the detected edge is the same or very similar to the reference image. Otherwise, when is closer to 0, there is a high difference between the detected edge and the reference image. 

In Table 10, is possible to appreciate the FOM values obtained with the synthetic images, using the detectors MG, SI + MG, IT2SI + MG, respectively. Comparing the obtained results, we can conclude that for this database, the GT2 FSI + MG provides better results, in most of the images than the traditional MG and IT2 SI + MG. 

After evaluating the BSDS database with the edge detectors, the obtained images can be appreciated in Table 11. The image number is in the first column. The images obtained using the aggregation operator based on the morphological gradient with the traditional Sugeno integral are presented in the second column. The images produced after applying the Sugeno integral with the operators of the fuzzy logic type-2 are shown in the third column. We can appreciate the images in which the proposed method GT2 FSI was implemented in the fourth column; in this column, the FD assigned at each gradient was 0.2. The last column presents the images after using the GT2 FSI, but in this case, the FD associated at each gradient was 0.7. It is possible to appreciate visually that the images present more information on the edges that the obtained with other methods and other FDs.

In Table 12, the FOM metrics of the BSDS database obtained after performing experiments with diverse edge detectors can be visualized. The database used for these tests differs from the previous one, since it contains real images of animals, landscapes or other things. However, this database also has reference images.

To have a better point of comparison, the Morphological gradient and Sobel detectors were also applied. In the obtained results, it can be observed that due to the nature of the information present in the images, the edge detection process turns out to be more complicated than in the synthetic image database, so that, in most cases, the calculated values with the metric are very low. However, they serve as a point of comparison for evaluating the detectors used.

The 16 images were evaluated with the MG detectors, Sobel, SI + MG, IT2 SI + MG, and GT2 FSI + MG. In the case of the aggregators using measures, FD = 0.2 was associated with the gradients of each integrator; according to the FOM metric, Sobel was better in three images, SI + MG in two, IT2 SI + MG, in three, while that GT2 FSI + MG achieved better results in eight images; which this last represents the 50 percent of the image database.

In most cases, the task of edge detection is improved when a traditional edge detection method (MG or Sobel) is combined with some other technique, for example an aggregation operator that uses measures or a fuzzy system [9,10,12,13,14] especially when the ideal parameters for the measures or MF used are known. In this case, the tests carried out with the proposed method were made by assigning fuzzy densities of 0.2 to each of the gradients calculated in the four directions. However, it does not mean that these densities are appropriate because they are arbitrarily established, so it is expected that by varying the FD the results obtained when calculating the FOM metric will improve. 

## 7. Conclusions and Future Works

In summary, this paper presents a fuzzy approach for edge detection applying the general type-2 fuzzy Sugeno integral as a method to aggregate the image gradients; this provides a more robust system because the images are viewed as fuzzy information and this gives the advantage of modeling the uncertainty contained in the data.

In Section 6, we presented a comparative analysis of the different used methods to aggregate the image gradients, such as the traditional Sugeno integral, IT2 SI, and the GT2 FSI. The results showed that the GT2 FIS is suitable to combine this type of information; above all to improve edge detection tasks based on gradients.

According to the results shown in Table 10 and Table 12, we can notice that edges of the image were better in most cases after applying the GT2 FSI + MG operator or working with fuzzy measures. However, it is necessary to perform more tests varying the fuzzy densities designated for each information sources, as well as the parameters of the general fuzzy system to verify if the results can be improved even more.

Operators that use fuzzy measures manage degrees of membership to each of the information sources, which allows managing various levels of uncertainty. Therefore, when using this type of aggregation operators, different alternatives can be explored when solving problems, so it is interesting to apply fuzzy measurements to several applications to explore a broader field of action, as in this case in the edges detectors.

We envision that the fuzzy densities associated with each of the information sources are optimal for the system and assigned automatically since they play an essential role in obtaining a solution with the proposed operator. Therefore, as future work, it is possible to implement optimization techniques with the purpose of finding the optimal values for the fuzzy densities and the parameters of the GT2 MFs; all this is performed in order to improve the proposed system and to take the most advantages of the aggregation operator. In this research, we present an approximation of the combination of the general fuzzy sets with the Sugeno integral; however, it is necessary to consider whether there are other ways to combine the proposed methods.

## Figures and Tables

**Table 1:** Fuzzy densities generated by each alpha cut.

	$M_{iL\alpha_{i}}\left(x_{i}\right)$					$M_{iR\alpha_{i}}\left(x_{i}\right)$				
x_i_\α_i_	0.2	0.4	0.6	0.8	0.99	0.2	0.4	0.6	0.8	0.99
**x_1_**	0.0068	0.0767	0.1312	0.1856	0.2692	0.5765	0.5066	0.4521	0.3977	0.3142
**x_2_**	0.2060	0.2680	0.3162	0.3644	0.4384	0.7106	0.6487	0.6005	0.5523	0.4783
**x_3_**	0.0001	0.0241	0.0708	0.1174	0.1890	0.4525	0.3926	0.3459	0.2992	0.2276
**x_4_**	0.3056	0.3636	0.4087	0.4538	0.5230	0.7777	0.7198	0.6746	0.6296	0.5603

**Table 2:** Gradients generated by each alpha cut.

	$D_{L\alpha_{i}}\left(x_{i}\right)$					$D_{R\alpha_{i}}\left(x_{i}\right)$				
x_i_\α_i_	0.2	0.4	0.6	0.8	0.99	0.2	0.4	0.6	0.8	0.99
**x_1_**	0	0.0282	0.0754	0.1227	0.1952	0.4620	0.4013	0.3541	0.3068	0.2343
**x_2_**	0	0	0.0374	0.0797	0.1447	0.3839	0.3295	0.2871	0.2448	0.1798
**x_3_**	0	0.0563	0.1077	0.1591	0.2381	0.5284	0.4624	0.4109	0.3595	0.2806
**x_4_**	0	0.0241	0.0708	0.1174	0.1890	0.4525	0.3926	0.3459	0.2992	0.2276

**Table 3:** Left and right lambda values generated by each alpha cut.

$\lambda_{L\alpha_{i}}$					$\lambda_{R\alpha_{i}}$				
0.2	0.4	0.6	0.8	0.99	0.2	0.4	0.6	0.8	0.99
−1.74 × 10^−16^	1.4891	0.2495	−0.2916	−0.6745	−0.9829	−0.9634	−0.9375	−0.8964	−0.7819

**Table 4:** Fuzzy measures calculated by each alpha cut.

	$\mu_{L\alpha_{i}}$					$\mu_{R\alpha_{i}}$				
x_i_\α_i_	0.2	0.4	0.6	0.8	0.99	0.2	0.4	0.6	0.8	0.99
**x_1_**	0.0001	0.0241	0.0708	0.1174	0.1890	0.4525	0.3926	0.3459	0.2992	0.2276
**x_2_**	0.0069	0.1036	0.2043	0.2967	0.4239	0.7726	0.7076	0.6514	0.5903	0.4859
**x_3_**	0.3126	0.5233	0.6338	0.7112	0.7974	0.9597	0.9367	0.9141	0.8867	0.8333
**x_4_**	0.5186	1	1	1	1	1	1	1	1	1

**Table 5:** Min values generated of the information sources with respect to the fuzzy measures.

	$\mu_{L\alpha_{i}}$					$\mu_{R\alpha_{i}}$				
x_i_\α_i_	0.2	0.4	0.6	0.8	0.99	0.2	0.4	0.6	0.8	0.99
**x_1_**	0	0.0241	0.0708	0.1174	0.189	0.4525	0.3926	0.3459	0.2992	0.2276
**x_2_**	0	0	0.0374	0.0797	0.1447	0.3839	0.3295	0.2871	0.2448	0.1798
**x_3_**	0	0.0563	0.1077	0.1591	0.2381	0.5284	0.4624	0.4109	0.3595	0.2806
**x_4_**	0	0.0241	0.0708	0.1174	0.189	0.4525	0.3926	0.3459	0.2992	0.2276

**Table 6:** Intervals generated by each alpha cut.

α_i_	Left Interval	Right Interval
**0.2**	0	0.3839
**0.4**	0	0.3295
**0.6**	0.0374	0.2871
**0.8**	0.0797	0.2448
**0.99**	0.1447	0.1798

**Table 7:** Synthetic images database together with their reference images.

*Image Number*	*Synthetic Image*	*Reference Image*
1	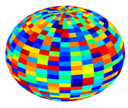	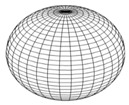
2	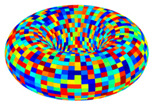	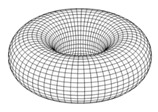
3	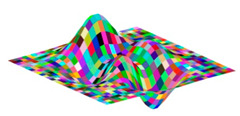	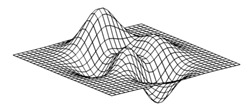
4	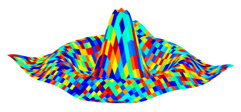	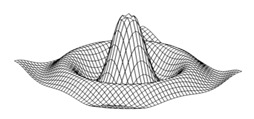
5	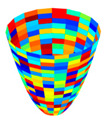	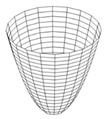

**Table 8:** A sample of the benchmark database of image segmentation and boundary detection (BSDS)l database with the original images and their reference images.

Image Number	Original Image	Reference Image	Image Number	Original Image	Reference Image
1	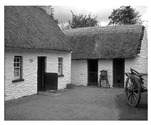	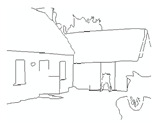	9	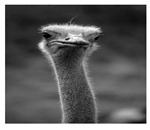	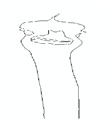
2	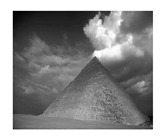	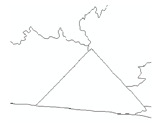	10	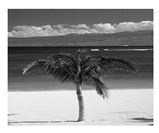	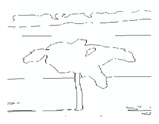
3	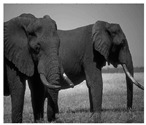	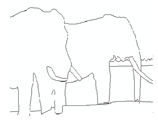	11	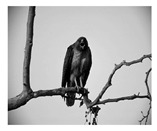	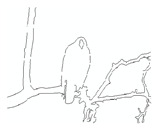
4	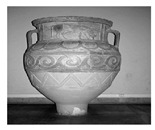	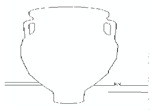	12	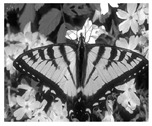	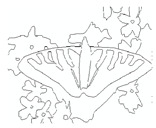
5	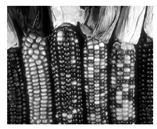	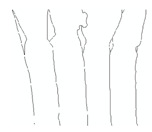	13	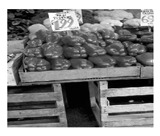	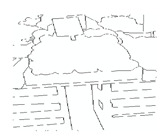
6	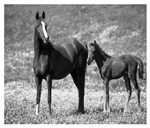	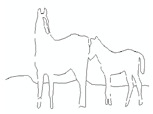	14	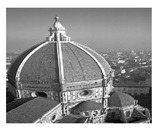	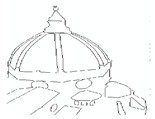
7	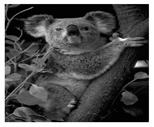	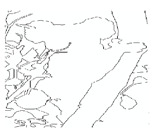	15	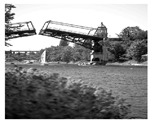	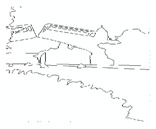
8	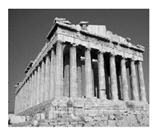	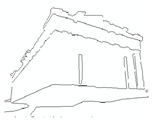	16	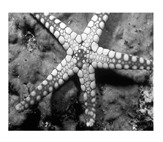	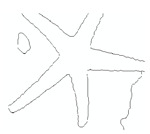

**Table 9:** Edge detectors in synthetic images.

Image Number	MG	SI + MG	IT2 SI + MG	GT2 FSI + MG	FD
1	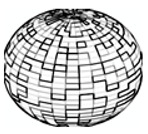	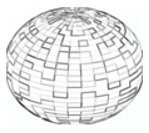	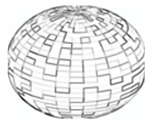	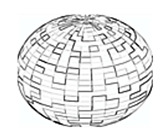	0.6
2	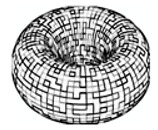	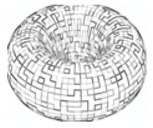	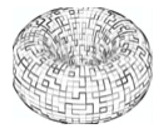	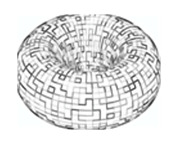	0.6
3	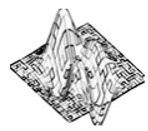	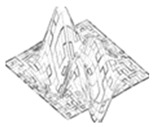	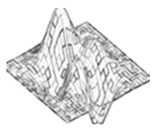	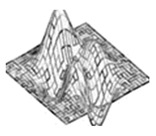	0.2
4	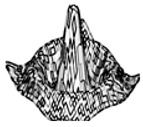	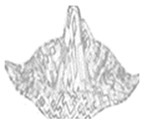	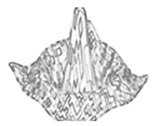	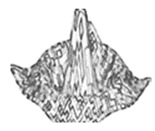	0.2
5	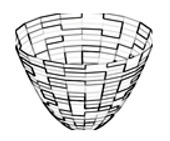	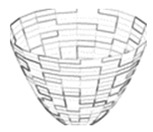	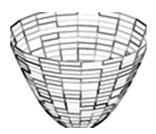	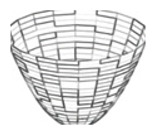	0.2

**Table 10:** "Figure of merit" (FOM) metric calculated using different edge detectors in synthetic images.

Image Number	MG	SI + MG	IT2 SI + MG	GT2 FSI + MG
1	0.8199	0.9408	0.9465	**0.9503**
2	0.8744	0.9477	0.9503	**0.9582**
3	0.8704	0.8841	0.8964	**0.8981**
4	0.9197	0.9199	0.9204	**0.9218**
5	0.8621	0.8812	0.9202	**0.9286**

**Table 11:** The BSDS image database after implementing edge detectors.

Image Number	Sugeno Integral (FD = 0.2)	IT2 SI + MG (FD = 0.2)	GT2 FSI + MG (FD = 0.2)	GT2 FSI + MG (FD = 0.7)
1	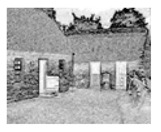	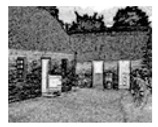	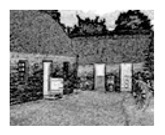	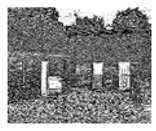
2	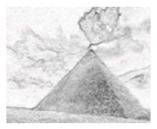	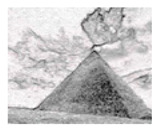	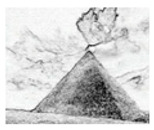	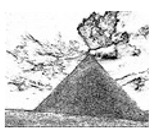
3	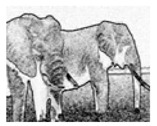	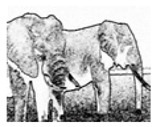	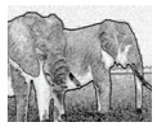	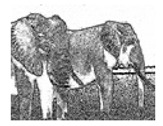
4	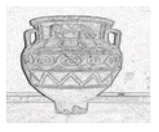	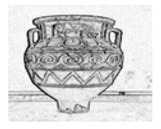	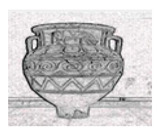	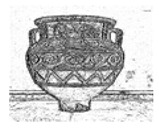
5	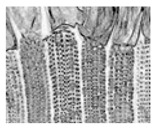	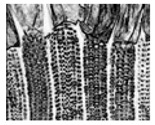	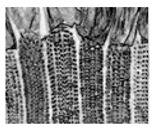	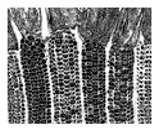
6	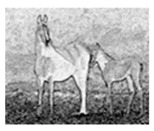	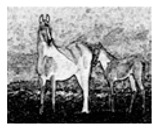	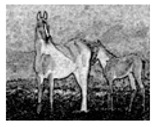	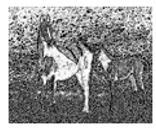
7	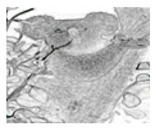	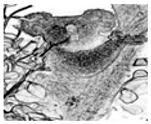	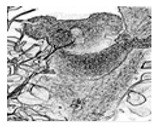	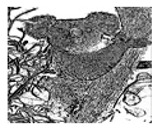
8	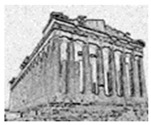	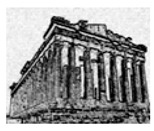	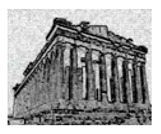	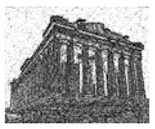
9	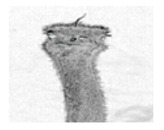	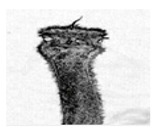	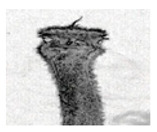	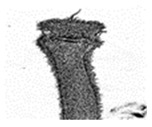
10	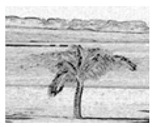	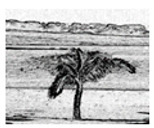	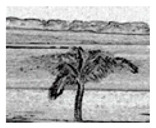	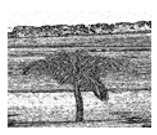
11	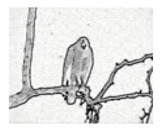	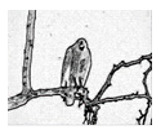	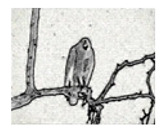	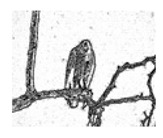
12	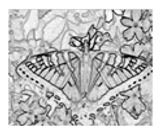	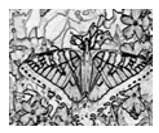	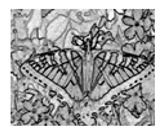	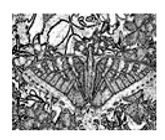
13	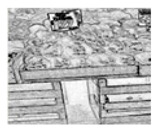	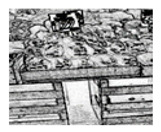	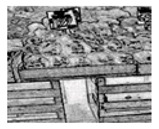	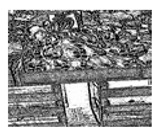
14	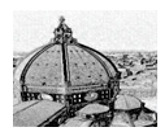	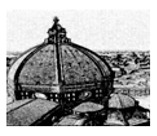	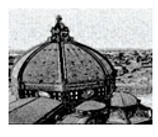	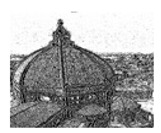
15	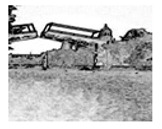	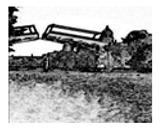	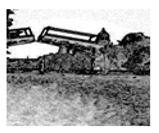	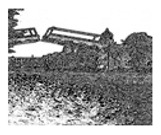
16	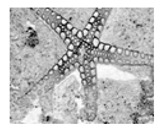	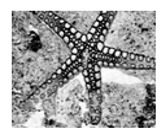	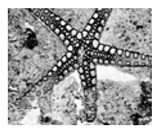	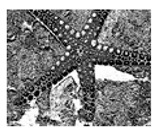

**Table 12:** FOM metric obtained after using edge detectors in the BSDS database.

Image Number	Edge Detectors				
	MG	SOBEL	SI + MG (FD = 0.2)	IT2 SI + MG (FD = 0.2)	GT2 FSI + MG (FD = 0.2)
1	0.3424	0.3944	0.3624	0.3974	**0.4029**
2	0.1406	0.1476	0.1506	0.1488	**0.1634**
3	0.3387	**0.4621**	0.3587	0.4061	0.4223
4	0.2225	0.2767	0.2425	**0.3533**	0.3356
5	0.1053	0.0968	0.1035	0.0986	**0.1037**
6	0.2133	0.2438	0.2333	0.2412	**0.2517**
7	0.3792	**0.4454**	0.3992	0.4006	0.4276
8	0.2755	0.3071	0.2955	0.3098	**0.3098**
9	0.2144	0.2406	0.2344	0.2378	**0.2428**
10	0.3837	0.4422	0.4037	0.4471	**0.4498**
11	0.7551	0.7727	0.6693	**0.7751**	0.7429
12	0.5492	0.5397	**0.5492**	0.544	0.4282
13	0.4787	0.4719	**0.4987**	0.4948	0.4876
14	0.401	0.4087	0.411	**0.4259**	0.4038
15	0.4508	**0.479**	0.4308	0.4211	0.3944
16	0.1809	0.1932	0.1921	0.1954	**0.1981**
